# Von Willebrand factor medates distinct mechanisms of vegetation formation in streptococcus mutans-induced endocarditis: comparisons between damage-induced and inflammation-induced mouse models

**DOI:** 10.3389/fcvm.2026.1490579

**Published:** 2026-08-06

**Authors:** Anni Li, Junwei Qian, Kangshuai Zhou, Yinuo Yuan, Senlin Ma, Dian Zhang, Xiaofei Jiang, Mingquan Chen

**Affiliations:** 1 Department of Infectious Diseases, Shanghai Key Laboratory of Infectious Diseases and Biosafety Emergency Response, National Medical Center for Infectious Diseases, Huashan Hospital, Fudan University, Shanghai, China; 2 Department of Emergency Medicine, Huashan Hospital, Fudan University, Shanghai, China

**Keywords:** ICAM 1, IL-17A, infective endocarditis, mouse model, streptococcus mutans, VWF (von willibrand factor)

## Abstract

Infective endocarditis (IE) is a severe cardiovascular disease characterized by the formation of vegetations on heart valves. This study investigates the role of von Willebrand Factor (VWF) in the pathogenesis of *Streptococcus mutans*-induced endocarditis using two distinct mouse models: damage-induced and inflammation-induced endocarditis. We employed histological analysis, immunofluorescence, scanning electron microscopy, and molecular techniques to elucidate the mechanisms of vegetation formation and the contribution of VWF in each model. Our results demonstrate that VWF is critical for vegetation formation in S. mutans-induced IE, with more pronounced effects in damage-induced models. VWF deficiency reduces valve colonization, and vegetation size, while supplementation restores these phenotypes. Mechanistically, VWF might upregulates IL-17A/ICAM-1 signaling to amplify inflammation and collagen deposition, which was validated by genetic knockout and antibody blockade. Furthermore, our study provides novel insights into the interplay between VWF and inflammatory responses during endocarditis progression, opening new avenues for understanding the complex pathophysiology of this life-threatening condition.

## Introduction

Infective endocarditis (IE) is a life-threatening cardiovascular disease characterized by the formation of vegetations on heart valves, most commonly affecting the aortic and mitral valves (1, 2). These vegetations consist of a complex amalgamation of platelets, fibrin, inflammatory cells, and colonizing bacteria (3). The incidence of IE has been increasing in recent years, particularly in developed countries, due to factors such as an aging population, increased use of invasive medical procedures, and the rise of antibiotic-resistant bacterial strains (4).

*Streptococcus mutans*, a gram-positive coccus typically associated with dental caries, has emerged as an important causative agent in IE, particularly in cases linked to poor oral hygiene (5). While traditionally considered an oral pathogen, *S. mutans* has demonstrated the ability to enter the bloodstream and colonize cardiac tissues, leading to endocarditis (6). The mechanisms by which this organism transitions from a commensal oral bacterium to a systemic pathogen capable of causing endocarditis are still not fully understood, highlighting the need for further research in this area (7).

The pathogenesis of IE involves an intricate interplay between the host's hemostatic and inflammatory responses and bacterial virulence factors (8). Upon entering the bloodstream, bacteria can adhere to damaged or inflamed endocardial surfaces, initiating a cascade of events that leads to vegetation formation. This process involves the activation of platelets, deposition of fibrin, and recruitment of inflammatory cells, ultimately creating a niche for bacterial proliferation and protection from host immune defenses (9).

Von Willebrand Factor (VWF), a large multimeric glycoprotein, plays a crucial role in platelet adhesion and aggregation at sites of vascular injury (10). Synthesized by endothelial cells and megakaryocytes, VWF is stored in Weibel-Palade bodies and *α*-granules, respectively, and is released upon cellular activation (11). In the context of vascular injury or inflammation, VWF acts as a bridge between exposed subendothelial collagen and platelets, facilitating platelet adhesion and subsequent thrombus formation (12).

Recent studies have suggested that VWF may also contribute to the formation and progression of endocarditic vegetations, although its precise role in different stages of the disease remains unclear (13, 14). The multifunctional nature of VWF, including its interactions with platelets, endothelial cells, and various plasma proteins, suggests that it may play a complex role in the pathogenesis of IE beyond its well-established hemostatic functions (15).

To better understand the mechanisms underlying vegetation formation in IE, researchers have developed various animal models that simulate different aspects of the disease process (16). Two commonly used approaches are the damage-induced model, which involves mechanical injury to the valve surface, and the inflammation-induced model, which relies on the induction of a systemic inflammatory response (17, 18). These models allow for the investigation of distinct pathogenic mechanisms and provide valuable insights into the complex interplay between host factors and bacterial virulence in the development of IE.

Our clinical data highlighted S. mutans as an underrecognized high-risk IE pathogen with elevated embolic complications. In this study, we aimed to investigate the distinct mechanisms of vegetation formation in damage-induced and inflammation-induced S. mutans endocarditis models in mice, with a particular focus on the role of VWF. We hypothesized that the contribution of VWF to vegetation development would differ between these two models, reflecting the varied pathogenic processes involved. By elucidating these aspects, we aimed to provide new insights into the pathogenesis of S. mutans endocarditis and identify potential therapeutic targets for prevention and treatment strategies.

Interleukin-17A (IL-17A) is a proinflammatory cytokine that plays a critical role in host defense against microbial infections and the pathogenesis of inflammatory diseases, including endocarditis (19). It promotes neutrophil recruitment and tissue inflammation by upregulating adhesion molecules such as intercellular adhesion molecule-1 (ICAM-1). ICAM-1, expressed on endothelial cells, mediates leukocyte adhesion and transmigration, which are key steps in the development of infective endocarditis (IE) lesions (vegetations) (20). Von Willebrand factor (VWF), a glycoprotein involved in hemostasis and inflammation, has been implicated in endothelial activation and leukocyte recruitment, but its interaction with IL-17A and ICAM-1 in S.mutans-induced IE remains unclear.

## Materials and methods

### Animal models

All animal experiments were conducted in accordance with institutional guidelines and approved by the local ethics committee. Eight- to ten-week-old male C57BL/6J mice (wild-type) and VWF knockout mice on a C57BL/6J background were used in this study. Both wild-type and VWF knockout mice were obtained from Southern Model Organisms Center (Nanjing, China). The VWF knockout mice were generously provided by Dr. Denis Wagner (Harvard Medical School) and have been previously characterized (21). Mice were housed in a specific pathogen-free facility with a 12-hour light/dark cycle and *ad libitum* access to food and water. Temperature and humidity were maintained at 22 ± 2 °C and 50 ± 10%, respectively. All procedures were performed under sterile conditions to minimize the risk of contamination.

### Induction of endocarditis models

Damage-induced model: Mice were anesthetized with isoflurane (2%–3% for induction, 1%–2% for maintenance) in oxygen. A small incision was made in the right side of the neck to expose the right carotid artery. A polyethylene catheter (PE-01, inner diameter 0.127 mm, outer diameter 0.254 mm) was inserted through the right carotid artery and advanced until it reached the aortic valve. Correct positioning was confirmed by feeling resistance and observing a slight pulsation of the catheter. The catheter was left in place for half of an hour to induce mechanical damage to the valve surface. Twenty-four hours after catheter removal, mice received an intravenous injection of 10^7 CFU of *S. mutans* (ATCC 25175) in 500 μL of sterile saline via the tail vein. The bacterial inoculum was prepared from an overnight culture in brain-heart infusion broth, washed twice in sterile saline, and adjusted to the appropriate concentration using spectrophotometry and confirmed by plate counting. Control (sham) mice underwent the same surgical procedures or received sterile saline injections without bacterial inoculation.

Inflammation-induced model: A catheter was inserted into the heart, and within 5 min of catheterization, histamine solution (50 mM) was infused at a rate of 10 μL/min. After infusion, the catheter was removed, the carotid artery was ligated, and the skin was sutured. Bacterial inoculation was performed via tail vein injection 24 h post-surgery. For the control group, an equal volume of sterile saline was infused into the heart instead of histamine, followed by bacterial inoculation via tail vein injection (22).

For each experimental group, a minimum of 8 mice were used to ensure statistical power, with additional animals included to account for potential losses during the procedure or follow-up period.

### Histological analysis

Mice were euthanized at 24 h, 3 days, or 5 days post-induction using CO2 inhalation followed by cervical dislocation. Hearts were rapidly excised, rinsed in cold phosphate-buffered saline (PBS), and fixed in 4% paraformaldehyde for 24 h at 4 °C. Following fixation, the hearts were processed through a series of graded ethanol solutions and xylene before being embedded in paraffin. Serial sections (5 μm thickness) were cut using a rotary microtome (Leica RM2255) and mounted on glass slides.

For general morphology, sections were stained with hematoxylin and eosin (H&E) using standard protocols. Briefly, sections were deparaffinized in xylene, rehydrated through graded alcohols, stained with Harris hematoxylin for 5 min, differentiated in 1% acid alcohol, and counterstained with eosin Y for 2 min. For bacterial visualization, Gram staining was performed using a commercial kit (Sigma-Aldrich) according to the manufacturer's instructions.

Slides were examined under a light microscope (Olympus BX51) equipped with a digital camera (Olympus DP72). Images were captured at various magnifications (40x, 100x, 400x) to provide both overview and detailed views of the valve structures and vegetations. For each animal, multiple sections were analyzed to ensure comprehensive evaluation of the valve morphology and vegetation characteristics.

### Immunofluorescence analysis

For immunofluorescence studies, hearts were fixed in 4% paraformaldehyde for 24 h, dehydrated through a graded ethanol series, cleared in xylene, and embedded in paraffin. Paraffin sections (5 µm thickness) were prepared using a microtome (Leica RM2235) and mounted on positively charged glass slides.

Sections were deparaffinized in xylene and rehydrated through a graded ethanol series to distilled water. Antigen retrieval was performed by heating the sections in citrate buffer (pH 6.0) for 20 min in a pressure cooker. After cooling to room temperature, sections were washed in PBS and permeabilized with 0.1% Triton X-100 in PBS for 10 min.

Sections were blocked with 5% bovine serum albumin (BSA) in PBS for 1 h at room temperature to minimize non-specific binding. Primary antibodies were diluted in 1% BSA/PBS and incubated overnight at 4 °C in a humidified chamber. The following primary antibodies were used: rat anti-mouse CD41 (1:100, Abcam), rabbit anti-mouse VWF (1:200, Abcam), goat anti-mouse fibrinogen (1:100, Abcam), rat anti-mouse IL-17A (1:100, BioLegend), rat anti-mouse CD31 (1:100, BD Biosciences), and Armenian hamster anti-mouse ICAM-1 (1:100, BD Biosciences).

After washing three times with PBS, sections were incubated with appropriate fluorochrome-conjugated secondary antibodies (all from Invitrogen, 1:500 dilution) for 1 h at room temperature in the dark. Secondary antibodies included Alexa Fluor 488-conjugated goat anti-rabbit IgG, Alexa Fluor 555-conjugated goat anti-rat IgG, Alexa Fluor 647-conjugated donkey anti-goat IgG, and Alexa Fluor 647-conjugated goat anti-Armenian hamster IgG. Nuclei were counterstained with DAPI (1 µg/mL, Sigma-Aldrich) for 5 min.

Slides were mounted using ProLong Gold Antifade Mountant (Invitrogen) and sealed with nail polish. Images were acquired using a confocal laser scanning microscope (Leica TCS SP8) equipped with 405 nm, 488 nm, 552 nm, and 638 nm laser lines. For each sample, at least five random fields were imaged using a 63× oil-immersion objective. Z-stack images were acquired to ensure complete capture of the three-dimensional structure of the vegetations.

Image analysis was performed using ImageJ software (NIH). Colocalization of different markers was quantified using the Coloc 2 plugin, and the intensity of staining was measured using the integrated density function. For each marker, the mean fluorescence intensity was calculated from at least three independent experiments.

### Scanning electron microscopy

For scanning electron microscopy (SEM) analysis, valve tissues were fixed in 2.5% glutaraldehyde in 0.1 M sodium cacodylate buffer (pH 7.4) for 2 h at room temperature. After washing in cacodylate buffer, samples were post-fixed in 1% osmium tetroxide for 1 h, followed by dehydration through a graded ethanol series (30%, 50%, 70%, 90%, 100%×2).

Samples were then subjected to critical point drying using liquid CO2 in a Tousimis Samdri-790 critical point dryer. Dried specimens were mounted on aluminum stubs using conductive carbon tape and sputter-coated with a 5 nm layer of gold-palladium using a Cressington 208HR sputter coater.

Samples were examined using a JEOL JSM-7600F field emission scanning electron microscope operated at an accelerating voltage of 5 kV. For each sample, multiple areas were imaged at various magnifications (500x, 2,000x, 5,000x, 10,000x) to capture both overall valve topography and detailed surface features. Particular attention was paid to areas of vegetation formation, bacterial colonization, and platelet aggregation.

### Plasma VWF:Ag measurement

Blood samples were collected via cardiac puncture into citrate-containing tubes (3.8% sodium citrate, 9:1 vol/vol) at the time of euthanasia. Plasma was separated by centrifugation at 2,000×g for 15 min at 4 °C and stored in aliquots at −80 °C until analysis.

VWF:Ag levels were measured using a commercial enzyme-linked immunosorbent assay (ELISA) kit (Affinity Biologicals) according to the manufacturer's instructions. Briefly, 96-well plates were coated with rabbit anti-human VWF antibody, blocked with 3% BSA, and incubated with diluted plasma samples or standards. Bound VWF was detected using horseradish peroxidase-conjugated anti-human VWF antibody and developed with 3,3',5,5'-tetramethylbenzidine (TMB) substrate. Absorbance was measured at 450 nm using a microplate reader (BioTek Synergy H1).

VWF:Ag levels were expressed as a percentage of normal pooled mouse plasma, with each sample measured in duplicate. The intra-assay and inter-assay coefficients of variation were <5% and <10%, respectively.

### Quantitative RT-PCR

Total RNA was extracted from valve tissues using TRIzol reagent (Invitrogen) according to the manufacturer's protocol. RNA concentration and purity were assessed using a NanoDrop spectrophotometer (Thermo Fisher Scientific), and RNA integrity was verified by agarose gel electrophoresis.

Complementary DNA (cDNA) was synthesized from 1 μg of total RNA using SuperScript III Reverse Transcriptase (Invitrogen) with oligo(dT) primers. Quantitative PCR was performed using SYBR Green Master Mix (Applied Biosystems) on a StepOnePlus Real-Time PCR System (Applied Biosystems). The following primer pairs were used:

VWF: Forward 5'-GCCTCTACCAGTGAGGTTGA-3’, Reverse 5'-GTTCACAGCAGTGAGTGTGG-3’ GAPDH: Forward 5'-AGGTCGGTGTGAACGGATTTG-3', Reverse 5'-TGTAGACCATGTAGTTGAGGTCA-3'

PCR conditions were as follows: initial denaturation at 95 °C for 10 min, followed by 40 cycles of 95 °C for 15 s and 60 °C for 1 min. Melting curve analysis was performed to ensure specificity of amplification. VWF mRNA expression was normalized to GAPDH as an internal control using the 2^-*ΔΔ*Ct method. Each sample was run in triplicate, and the mean value was used for analysis.

### VWF manipulation experiments

VWF blocking: Wild-type mice were injected intraperitoneally with 50 μg (per mouse) of anti-VWF antibody (Dako) or control IgG 4 h before endocarditis induction. The dose and timing of antibody administration were based on previous studies demonstrating effective VWF inhibition in murine models. To ensure sustained VWF inhibition, additional doses of antibody were administered at 24 and 48 h post-induction.

VWF supplementation: VWF KO mice were injected intraperitoneally with Humate P (3U VWF:RCo/mouse, Humate-P, CSL Behring) or an equal volume of saline 4 h before bacterial inoculation (23).

This dosing regimen was designed to maintain physiological levels of VWF throughout the experimental period, based on the half-life of recombinant VWF in mice.

For both VWF blocking and supplementation experiments, animals were monitored closely for any signs of bleeding or thrombosis. Blood samples were collected at 24, and 72 and 120 h post-treatment to assess plasma VWF:Ag levels and confirm the effectiveness of the interventions.

### Cell culture

Human umbilical vein endothelial cells (HUVECs) were purchased from [source, e.g., American Type Culture Collection, ATCC] and cultured in endothelial cell growth medium [ECGM; (manufacturer, e.g., PromoCell)] supplemented with 10% fetal bovine serum [FBS; (manufacturer, e.g., Gibco)], 1% penicillin-streptomycin [100 U/mL penicillin and 100 μg/mL streptomycin; (manufacturer, e.g., Thermo Fisher Scientific)], and endothelial cell growth supplement [ECGS; (manufacturer, e.g., PromoCell)] at 37 °C in a humidified atmosphere containing 5% CO₂. Cells were used between passages 3 and 6 to ensure phenotypic stability.

For experimental treatments, HUVECs were seeded in 6-well plates at a density of 5 × 10⁵ cells/well and allowed to reach 80%–90% confluence. Prior to stimulation with *Streptococcus mutans* [S.mutans; (strain, e.g., UA159)], cells were serum-starved in ECGM containing 0.5% FBS for 12 h. S.mutans was cultured overnight in brain heart infusion (BHI) broth at 37 °C under anaerobic conditions, then washed twice with phosphate-buffered saline (PBS) and adjusted to a final concentration of 1 × 10⁸ colony-forming units (CFU)/mL using ECGM. Cells were treated with S.mutans for 24 h. For neutralization or supplementation experiments:

Cells were pretreated with anti-VWF neutralizing antibody [[clone, e.g., AVW-1], 10 μg/mL; [manufacturer, e.g., Abcam]] or isotype control IgG [10 μg/mL; (manufacturer, e.g., Abcam)] for 1 h before S.mutans exposure.

### Protein extraction

After treatment, HUVECs were washed twice with ice-cold PBS to remove non-adherent bacteria and culture medium. Total cellular proteins were extracted using RIPA lysis buffer ([composition: 50 mM Tris-HCl pH 7.4, 150 mM NaCl, 1% Triton X-100, 0.5% sodium deoxycholate, 0.1% SDS] supplemented with 1× protease inhibitor cocktail [manufacturer, e.g., Roche] and 1× phosphatase inhibitor cocktail [manufacturer, e.g., Sigma-Aldrich]). Cells were scraped on ice, transferred to microcentrifuge tubes, and incubated on ice for 30 min with gentle vortexing every 10 min. Lysates were centrifuged at 12,000 × g for 15 min at 4 °C to pellet cell debris, and the supernatant (total protein extract) was collected.

Protein concentration was determined using the BCA Protein Assay Kit [(manufacturer, e.g., Thermo Fisher Scientific)] according to the manufacturer's instructions, with bovine serum albumin (BSA) as the standard. Extracts were stored at −80 °C until Western blotting analysis.

### Western blotting

Equal amounts of protein (30 μg per sample) were denatured in 5× SDS loading buffer [(composition: 250 mM Tris-HCl pH 6.8, 500 mM dithiothreitol, 10% SDS, 0.5% bromophenol blue, 50% glycerol)] at 95 °C for 5 min, then separated by 10% sodium dodecyl sulfate-polyacrylamide gel electrophoresis (SDS-PAGE) at 120 V for 90 min. Proteins were transferred to polyvinylidene difluoride (PVDF) membranes [(manufacturer, e.g., Millipore)] at 300 mA for 2 h using a wet transfer system. Membranes were blocked with 5% non-fat dry milk in Tris-buffered saline with Tween-20 [TBST; (composition: 20 mM Tris-HCl pH 7.6, 150 mM NaCl, 0.1% Tween-20)] for 1 h at room temperature, then incubated overnight at 4 °C with primary antibodies against ICAM-1 ([dilution, e.g., 1:1,000; clone, e.g., 15.2], [manufacturer, e.g., Santa Cruz Biotechnology]) or GAPDH ([dilution, e.g., 1:5,000; clone, e.g., 6C5], [manufacturer, e.g., Millipore]) as a loading control. After three washes with TBST (10 min each), membranes were incubated with horseradish peroxidase (HRP)-conjugated secondary antibodies ([dilution, e.g., 1:5,000; anti-mouse or anti-rabbit IgG], [manufacturer, e.g., Jackson ImmunoResearch]) for 1 h at room temperature. Protein bands were visualized using an enhanced chemiluminescence (ECL) detection kit [(manufacturer, e.g., Thermo Fisher Scientific)] and imaged with a gel documentation system [(model, e.g., ChemiDoc XRS+; Bio-Rad)]. Band intensities were quantified using ImageJ software (National Institutes of Health, USA), and ICAM-1 expression was normalized to GAPDH.

### Statistical analysis

Data are presented as mean ± standard deviation (SD) unless otherwise stated. Statistical analyses were performed using GraphPad Prism 9 software. Normal distribution of data was assessed using the Shapiro–Wilk test. For comparisons between two groups, Student's *t*-test (for normally distributed data) or Mann–Whitney U test (for non-normally distributed data) was used. Multiple group comparisons were analyzed by one-way ANOVA followed by Tukey's *post hoc* test for normally distributed data, or by Kruskal–Wallis test followed by Dunn's *post hoc* test for non-normally distributed data.

Two-way ANOVA was used to assess the effects of time and treatment in the VWF manipulation experiments. Correlations between plasma VWF:Ag levels and vegetation size were evaluated using Pearson's correlation coefficient. Kaplan–Meier survival curves were compared using the log-rank test.

*P*-values < 0.05 were considered statistically significant. All statistical tests were two-tailed, and the level of significance was adjusted for multiple comparisons using the Bonferroni method where appropriate.

## Results

### Survival, disease incidence, and bacterial burden in endocarditis models

#### Dose effects of Streptococcus mutans inoculum

Murine survival at 5 days post-inoculation with S. mutans (ATCC 25175) decreased with increasing bacterial load: 83.3% (10⁶ CFU), 66.0% (10⁷ CFU), and 33.3% (10⁸ CFU). The 10⁷ CFU dose yielded the highest vegetation positivity (75.0%), compared to 20.0% (10⁶ CFU) and 50.0% (10⁸ CFU), and was thus selected for subsequent experiments.

#### Histamine concentration in inflammation-induced model

Inflammation-induced IE was induced via carotid artery infusion of histamine (50 mM, 100 mM, 200 mM) followed by 10⁷ CFU S. mutans challenge. At 5 days, survival was 60.0% in 50 mM and 100 mM groups (0% in 200 mM). Vegetation incidence was 0% (50 mM) vs. 50.0% (100 mM), identifying 100 mM as the minimal effective concentration.

#### Temporal dynamics in damage-induced IE

Survival declined from 90.0% (day 1) to 60.0% (day 5). IE incidence increased progressively: 33.3% (day 1), 50.0% (day 3), 66.7% (day 5). Bacterial burden in vegetations (log CFU/mm^3^) rose over time: 1.67 ± 0.57 (day 1), 2.05 ± 0.48 (day 3), 2.85 ± 0.58 (day 5).

#### Temporal dynamics in inflammation-induced IE

Survival remained stable at 60.0% from day 3 to 5. IE incidence was 37.5% (day 1), 50.0% (day 3), 66.7% (day 5). Bacterial burden (log CFU/mm^3^) was higher than in the damage model at early stages: 2.70 ± 0.20 (day 1), 2.67 ± 0.76 (day 3), 3.43 ± 0.46 (day 5).

### Histological characterization of endocarditis progression

**Figure 1:**
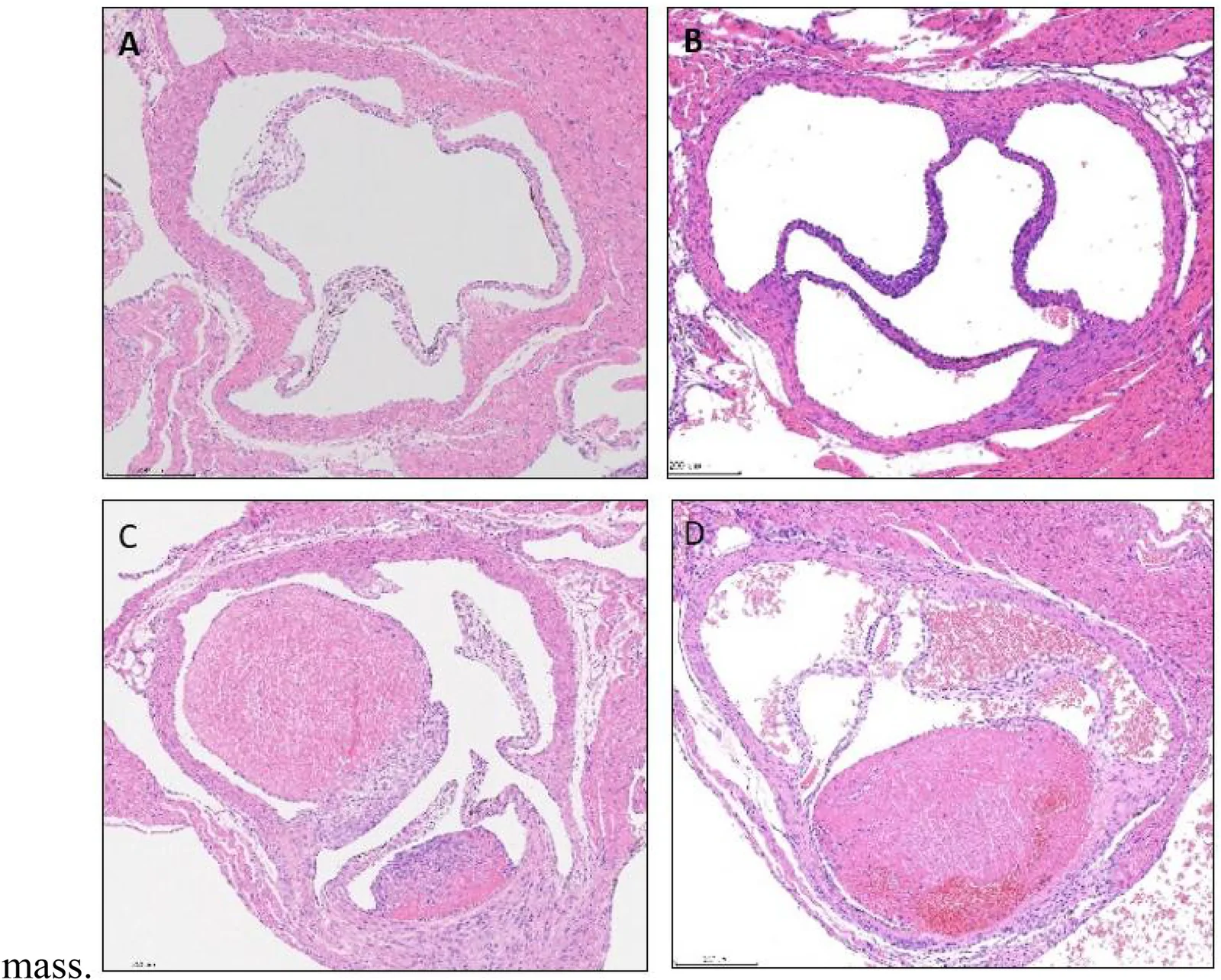
Histological analysis of aortic valves in damage- and inflammation-induced endocarditis models. **(A)** Damage control group: Aortic valve structure showing thin, well-organized leaflets. **(B)** Inflammation control group: Aortic valve with a large infiltration of inflammatory cells, characterized by thickening of valve leaflets. **(C)** Damage IE group: Aortic valve with advanced vegetation formation, exhibiting large, irregular masses attached to valve leaflets. **(D)** Inflammation IE group: Higher magnification of vegetation, revealing dense cellular infiltration and fibrin deposition within the vegetation.

In the damage-induced model, 85% of wild-type mice (17/20) developed endocarditis, defined by vegetation formation and bacterial colonization.Histological analysis of aortic valves revealed distinct patterns of vegetation formation in the damage-induced and inflammation-induced endocarditis models (Figure 1). In the damage control group, aortic valves showed generally normal structure with thin, well-organized leaflets (Figure 1A). The inflammation control group presented with thickening of valve leaflets and inflammatory cell infiltration in the perivalvular area (Figure 1B). In the damage IE group, large, irregular masses were observed attached to valve leaflets, indicating advanced vegetation formation (Figure 1C). Higher magnification examination of these vegetations in the inflammation IE group revealed dense cellular infiltration and fibrin deposition within the vegetation mass (Figure 1D).

The progression of vegetation formation differed between the damage-induced and inflammation-induced models. The damage-induced model showed rapid progression to advanced vegetation formation by day 5, as seen in Figure 1C. In contrast, the inflammation-induced model appeared to show a more gradual progression, with early changes visible in the control group (Figure 1B) and advanced changes, including dense cellular infiltration and fibrin deposition, observable in the IE group (Figure 1D).

**Figure 2:**
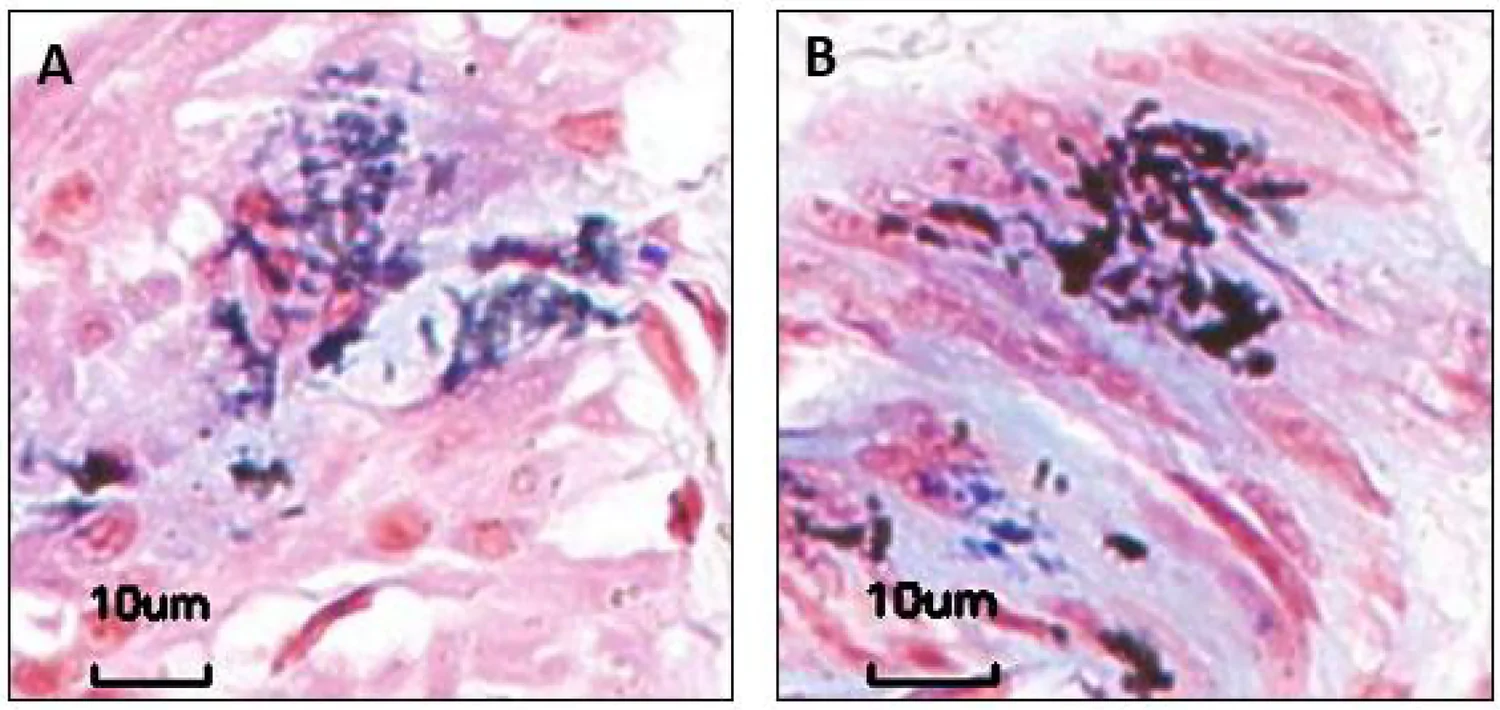
Gram staining of aortic valve sections demonstrating bacterial colonization. **(A)** Gram staining showing clusters of Gram-positive cocci (dark purple) within valve tissue, indicating bacterial colonization. Bacteria visible within the vegetation of the damage-induced IE model. **(B)** Higher magnification of bacterial colonies, demonstrating their integration within the valve structure and surrounding inflammatory cells. Bacteria visible within the vegetation of the inflammation-induced IE model.

Gram staining of aortic valve sections demonstrated bacterial colonization in both damage-induced and inflammation-induced IE models (Figure 2). In the damage-induced IE model, clusters of Gram-positive cocci (dark purple) were observed within the vegetation, indicating successful establishment of S. mutans infection (Figure 2A). Higher magnification images of the inflammation-induced IE model revealed the integration of bacterial colonies within the vegetation structure and surrounding inflammatory cells (Figure 2B). These findings confirm the presence of bacteria within the vegetations of both IE models, highlighting the successful establishment of infection in each case.

### Plasma VWF:Ag levels in endocarditis models

**Figure 3:**
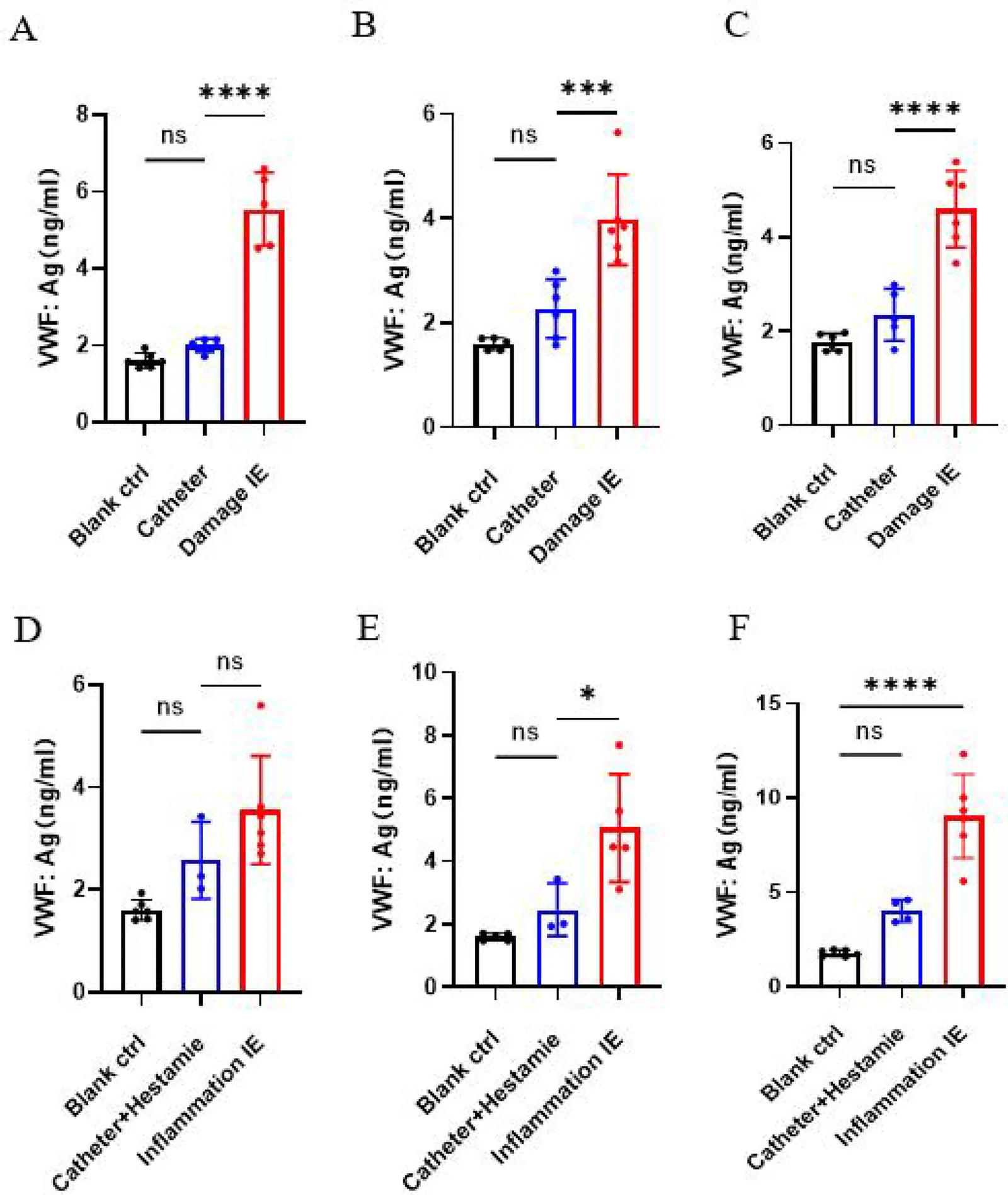
Plasma VWF:Ag levels in damage-induced and inflammation-induced endocarditis models. **(A)** Damage-induced model, 24 h post-induction: Significant increase in VWF:Ag levels compared to control. **(B)** Damage-induced model, 3 days post-induction: Sustained elevation of VWF:Ag levels. **(C)** Damage-induced model, 5 days post-induction: Further increase in VWF:Ag levels. **(D)** Inflammation-induced model, 24 h post-induction: No significant change in VWF:Ag levels. **(E)** Inflammation-induced model, 3 days post-induction: Moderate increase in VWF:Ag levels. **(F)** Inflammation-induced model, 5 days post-induction: Significant elevation of VWF:Ag levels.

Analysis of plasma VWF:Ag levels revealed distinct temporal patterns between the damage-induced and inflammation-induced endocarditis models (Figure 3). In the damage-induced model, a significant increase in VWF:Ag levels was observed as early as 24 h post-induction compared to control mice (Figure 3A). This elevation was sustained at day 3 (Figure 3B) and further increased by day 5 (Figure 3C).

In contrast, the inflammation-induced model showed a delayed and more gradual increase in plasma VWF:Ag levels. No significant change was observed at 24 h post-induction (Figure 3D). A moderate increase was noted by day 3 (Figure 3E), with significant elevation reaching levels comparable to the damage-induced model by day 5 (Figure 3F).

### VWF mRNA expression in aortic valve tissues

**Figure 4:**
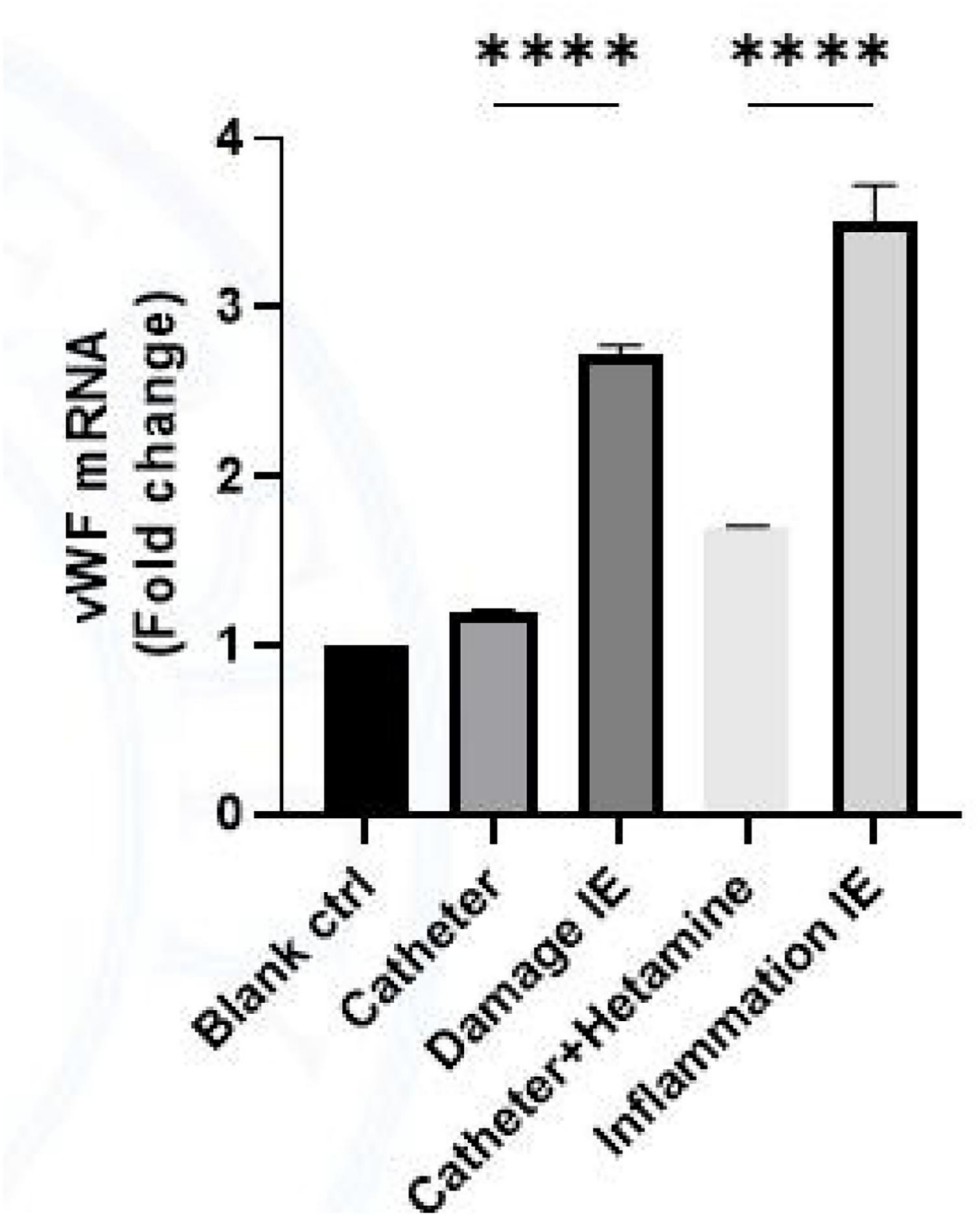
VWF mRNA expression in aortic valve tissues. Single panel showing relative VWF mRNA expression in control, damage-induced, and inflammation-induced endocarditis models, 5 days post-induction. Both models show significant upregulation of VWF mRNA compared to control.

Quantitative RT-PCR analysis of VWF mRNA expression in aortic valve tissues at day 5 post-induction revealed significant upregulation in both endocarditis models compared to control mice (Figure 4). The damage-induced model showed a more pronounced increase in VWF mRNA expression (approximately 5-fold) compared to the inflammation-induced model (approximately 3-fold).

### VWF deposition in aortic valves

**Figure 5:**
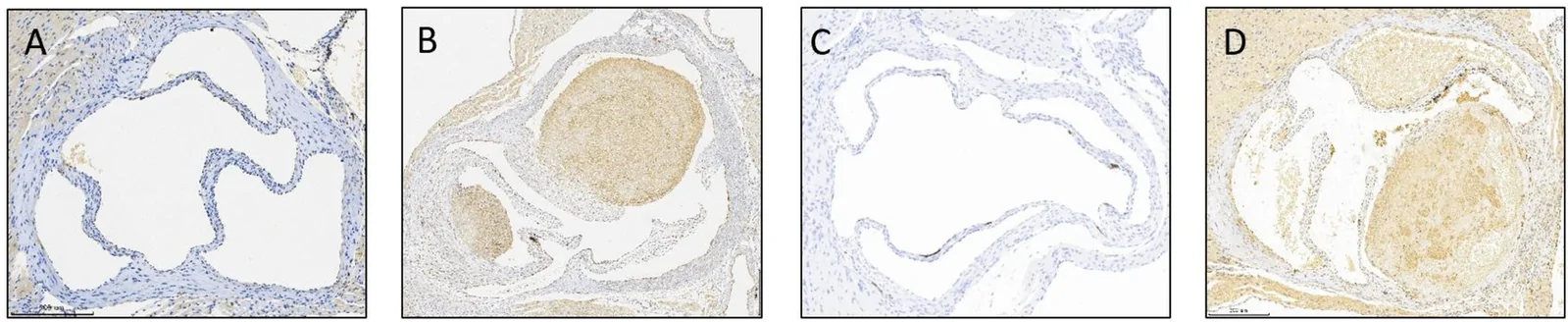
VWF deposition in aortic valves. **(A)** Sham surgery group showing minimal VWF staining (light brown). **(B)** Damage-induced endocarditis model demonstrating increased VWF deposition (dark brown) within valve tissue and vegetation. **(C)** Inflammation control group showing VWF slight staining at the edge of the valve. **(D)** Inflammation-induced endocarditis model exhibiting extensive VWF deposition throughout the valve and associated vegetation.

Immunohistochemical staining for VWF in aortic valve sections demonstrated distinct VWF deposition patterns across different groups, with increased deposition observed in both endocarditis models compared to the sham surgery group and the inflammation control group (Figure 5). The sham surgery group showed minimal VWF staining (light brown) in aortic valves (Figure 5A). In contrast, the damage-induced endocarditis model exhibited notably increased VWF deposition (dark brown) within the valve tissue and associated vegetations (Figure 5B). The inflammation control group, as a reference, displayed slight VWF staining primarily at the edge of the valve (Figure 5C). Most prominently, the inflammation-induced endocarditis model demonstrated extensive VWF deposition throughout the entire valve and its associated vegetation (Figure 5D).

### Ultrastructural analysis of early endocarditis lesions

**Figure 6:**
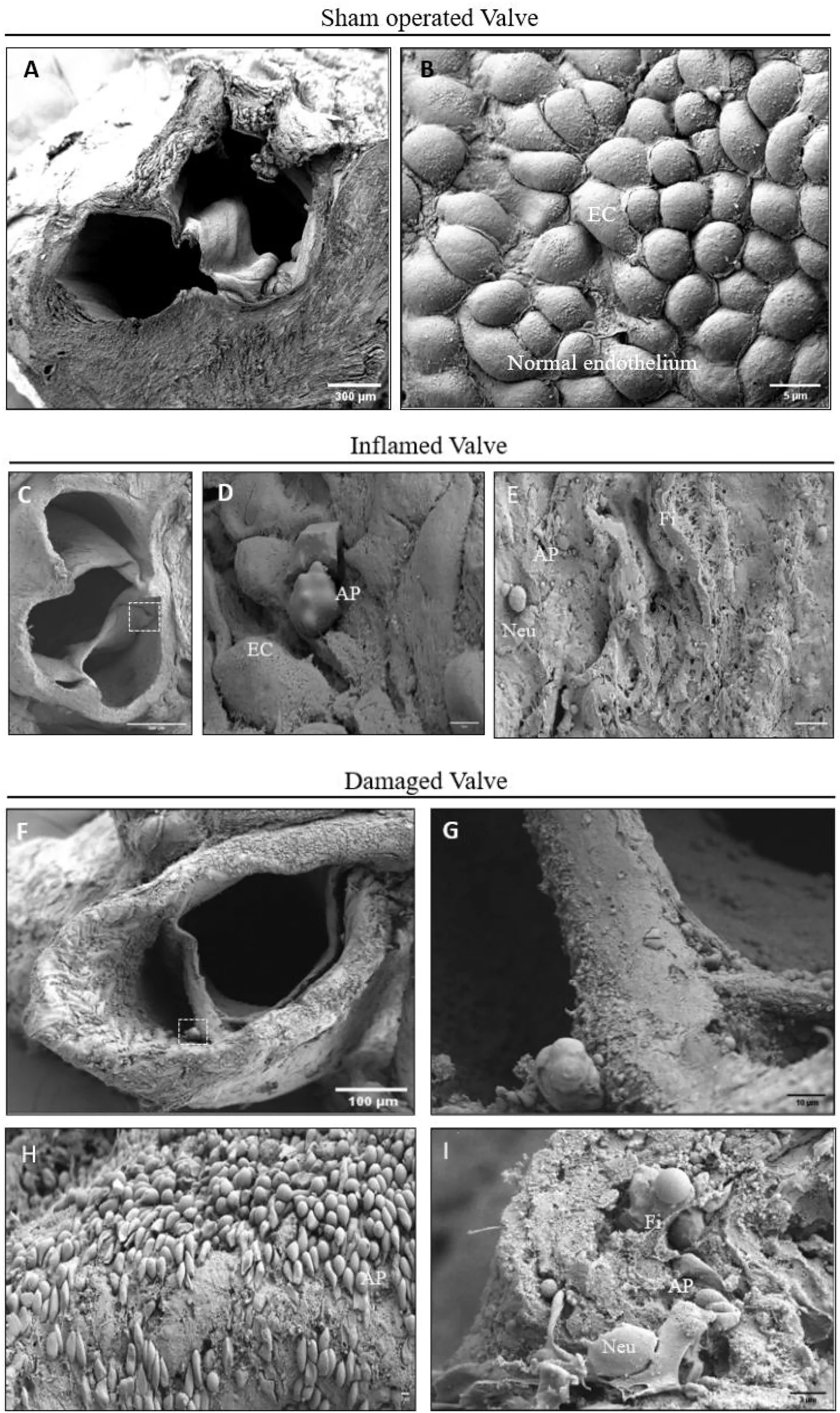
Scanning electron microscopy of early endocarditis lesions in valve models. Sham Surgery Group: (A) Low magnification image showing overall intact aortic valve surface with no significant attachments. (B) High magnification image revealing intact endothelium with no obvious lesions. Inflammation-Induced Model: (C) Early stage of S. mutans IE after inflammation induction, showing early lesions formation on the valve surface. (D, E) High magnification image showed activated platelets, leukocyte, and collagen fibers adhering to the valve surface. Damage-Induced Model: (F, G) Overview of damaged valve surface with significant endothelial disruption and disordered structure. (H) Detailed view of platelet aggregation on the aortic valve surface. (I) High magnification image of leukocyte adhesion to the damaged valve surface.

Scanning electron microscopy (SEM) of early endocarditis lesions revealed distinct ultrastructural changes in the different valve models (Figure 6). The sham surgery group showed intact aortic valve surfaces with no significant attachments at low magnification (Figure 6A). Higher magnification images revealed intact endothelium with no obvious lesions (Figure 6B).

In the inflammation-induced model, SEM analysis captured various stages of S. mutans infection and vegetation formation. Early stages showed fibrin network formation on the valve surface (Figure 6C), progressing to moderate stages with activated platelets and leukocytes adhering to the valve surface (Figure 6D). Advanced stages of inflammation were characterized by increased cellular aggregation on the valve (Figure 6E).

Detailed examination of the damage-induced model revealed significant endothelial disruption and disordered structure (Figure 6F). Higher magnification images showed thick platelet aggregation on the aortic valve (Figure 6G), collagen fibers adhering to the aortic valve surface (Figure 6H), and areas of leukocyte adhesion to the damaged valve surface (Figure 6I).

### Immunofluorescence analysis of wild-type mouse valve tissues

**Figure 7:**
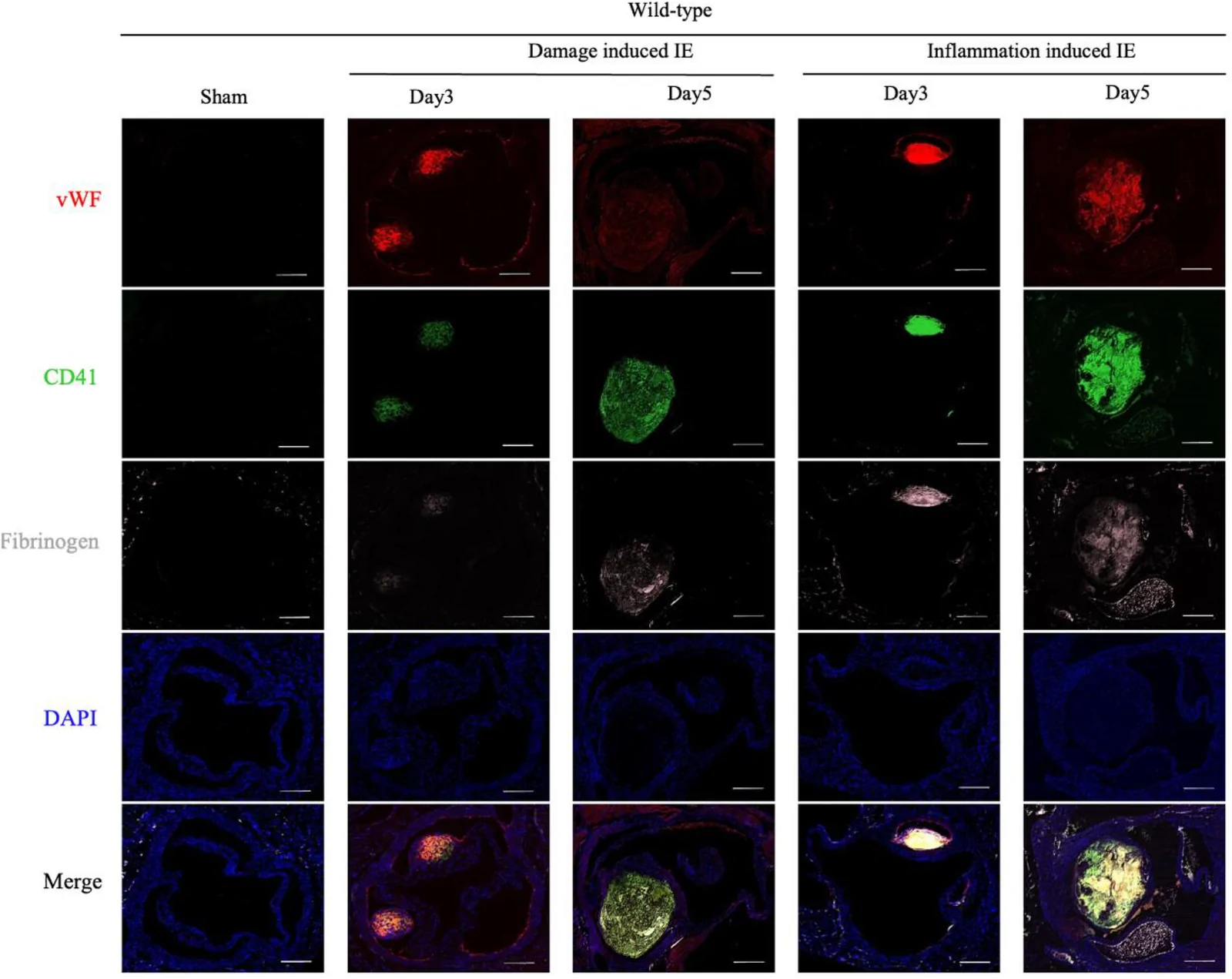
Immunofluorescence analysis of wild-type mouse valve tissues in different endocarditis models. Representative images of valve tissues from wild-type mice under sham conditions, damage-induced endocarditis (Day 3 and Day 5), and inflammation-induced endocarditis (Day 3 and Day 5). Tissues were stained for VWF (red), CD41 (green, platelet marker), fibrinogen (gray), and DAPI (blue, nuclear stain). Overlay images are shown in the bottom row. Both damage-induced and inflammation-induced endocarditis models demonstrate progressive formation of valve vegetations, characterized by co-localization of platelets, VWF, and fibrinogen. Scale bars: 100 μm.

Immunofluorescence analysis of wild-type mouse valve tissues provided insights into the spatial and temporal patterns of key components involved in vegetation formation (Figure 7). Tissues were stained for CD41 (platelet marker, red), VWF (green), fibrinogen (gray), and DAPI (nuclear stain, blue).

In the damage-induced model, co-localization of platelets, VWF, and fibrinogen was evident as early as day 3, with extensive vegetation formation observed by day 5. The inflammation-induced model showed a more gradual progression, with minimal co-localization at day 3 and moderate vegetation formation by day 5.

These findings corroborate the histological observations and highlight the differential kinetics of vegetation formation between the two models.

### Analysis of VWF knockout mice in endocarditis models

**Figure 8:**
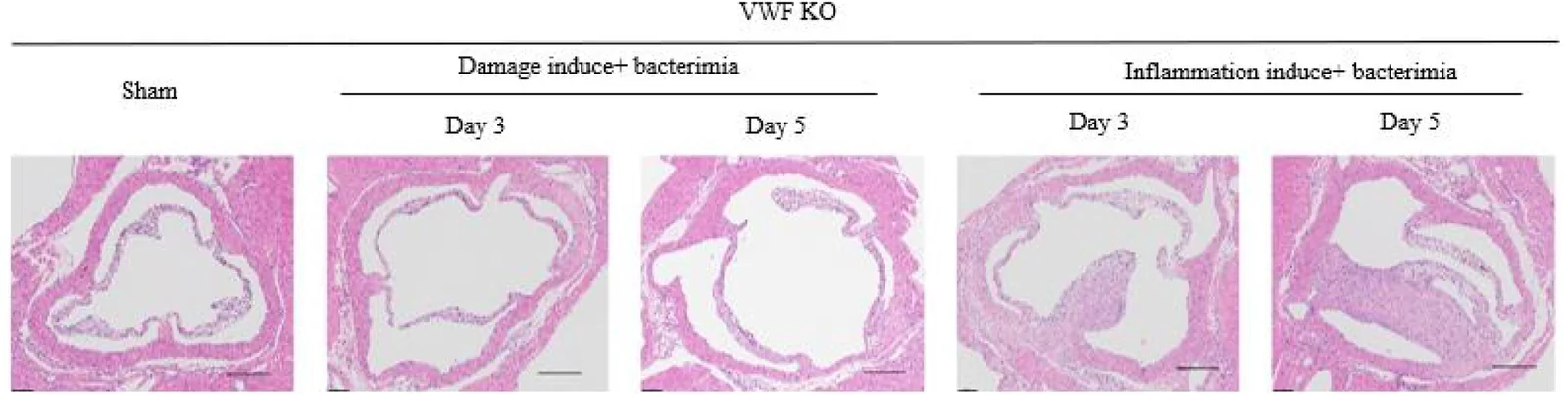
Histological analysis of valve tissues in VWF knockout mice under different endocarditis conditions. H&E staining of valve tissues from VWF knockout (KO) mice. Images show sham condition, damage-induced endocarditis (Day 3 and Day 5), and inflammation-induced endocarditis (Day 3 and Day 5). VWF KO mice exhibit minimal vegetation formation in the damage-induced model, while small new vegetations are observed in the inflammation-induced model, particularly at Day 5. Scale bars: 100 μm.

To further elucidate the role of VWF in vegetation formation, we examined valve tissues from VWF knockout (KO) mice under different endocarditis conditions (Figure 8). H&E staining revealed striking differences in vegetation formation between wild-type and VWF KO mice.

In the damage-induced model, VWF KO mice exhibited minimal vegetation formation even at day 5 post-induction. The inflammation-induced model showed small new vegetations in VWF KO mice, particularly at day 5, but these were considerably smaller than those observed in wild-type mice.

**Figure 9:**
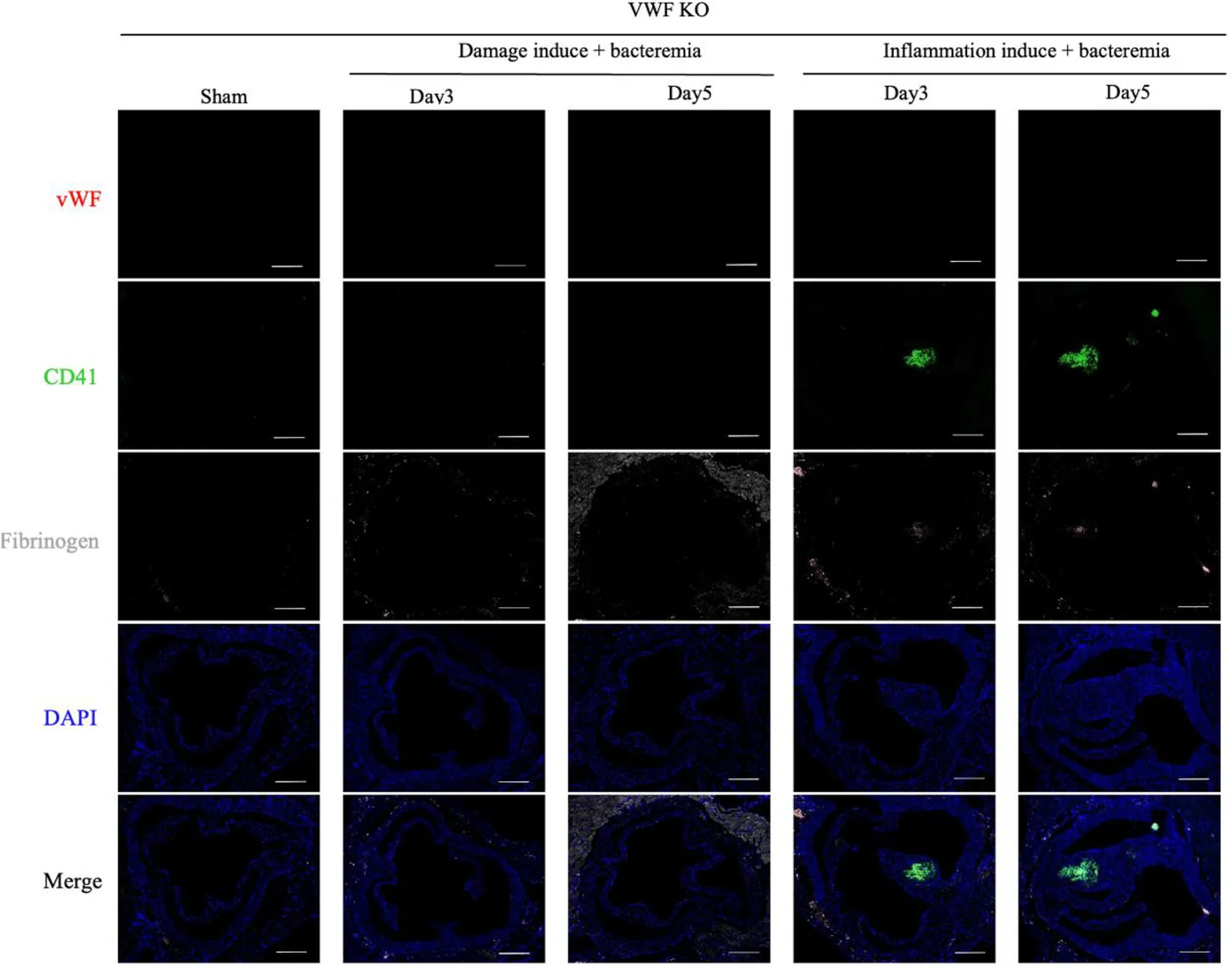
Detailed immunofluorescence analysis of valve tissues in VWF knockout mice during endocarditis progression. VWF knockout (KO) mice valve tissues were analyzed under sham conditions, damage-induced endocarditis (Day 3 and Day 5), and inflammation-induced endocarditis (Day 3 and Day 5). Tissues were stained for VWF (red, absent due to knockout), CD41 (green), fibrinogen (gray), and DAPI (blue). Overlay images are shown in the bottom row. Note the absence of substantial vegetations in the damage-induced model, but small new vegetations primarily composed of platelets (CD41-positive) in the inflammation-induced model. Scale bars: 100 μm.

Detailed immunofluorescence analysis of VWF KO mouse valve tissues (Figure 9) confirmed the absence of VWF staining and revealed reduced platelet aggregation and fibrinogen deposition compared to wild-type mice. In the damage-induced model, VWF KO mice showed minimal vegetation formation, with only small aggregates of platelets (CD41-positive) observed. The inflammation-induced model in VWF KO mice demonstrated small new vegetations primarily composed of platelets, but these were significantly reduced compared to wild-type counterparts.

### Effects of VWF manipulation on vegetation formation

**Figure 10:**
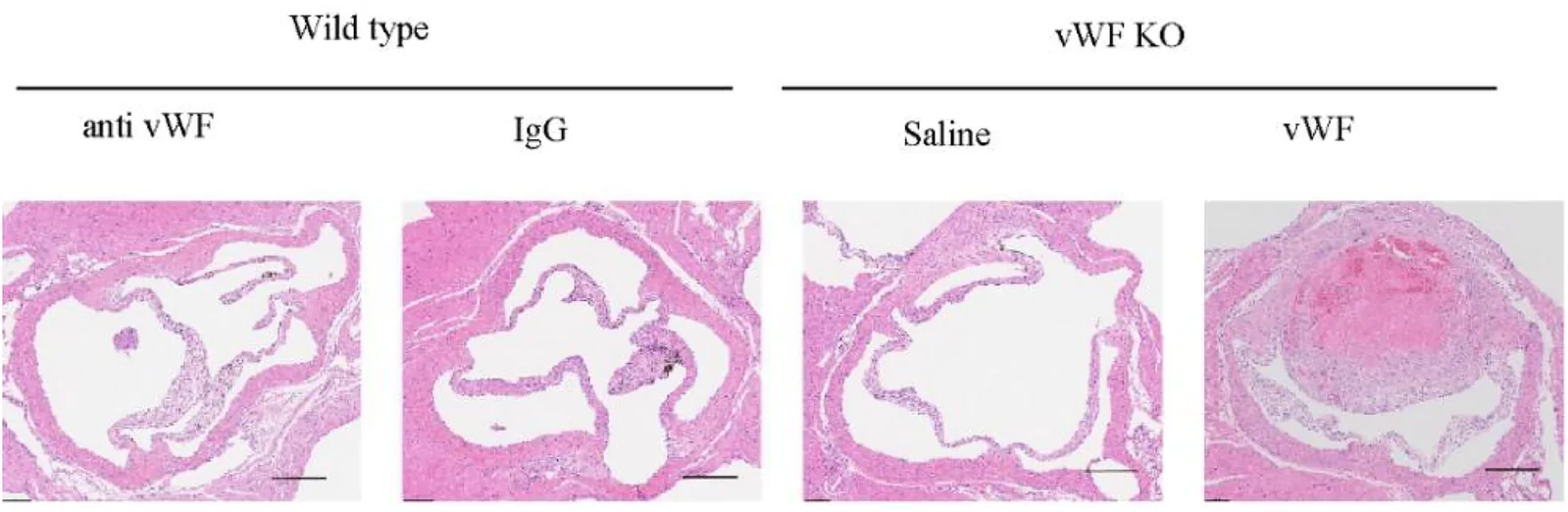
Effects of VWF manipulation on valve vegetation formation. H&E staining of valve tissues under different conditions. Wild-type mice treated with anti-VWF antibody, Wild-type mice treated with IgG control, VWF KO mice treated with saline, and VWF KO mice treated with VWF supplementation. VWF supplementation in VWF KO mice restores vegetation formation, visible as large masses on the valve surface. Conversely, VWF blocking in wild-type mice inhibits vegetation formation, resulting in relatively normal valve appearance. Scale bars: 100 μm.

To further investigate the causative role of VWF in vegetation formation, we performed VWF manipulation experiments (Figure 10). Wild-type mice treated with anti-VWF antibody showed inhibited vegetation formation, resulting in a relatively normal valve appearance (Figure 10A). This was in stark contrast to wild-type mice treated with control IgG, which developed substantial vegetations (Figure 10B).

Conversely, VWF KO mice treated with saline showed minimal vegetation formation (Figure 10C), while VWF KO mice receiving VWF supplementation exhibited restored vegetation formation, visible as large masses on the valve surface (Figure 10D).

**Figure 11:**
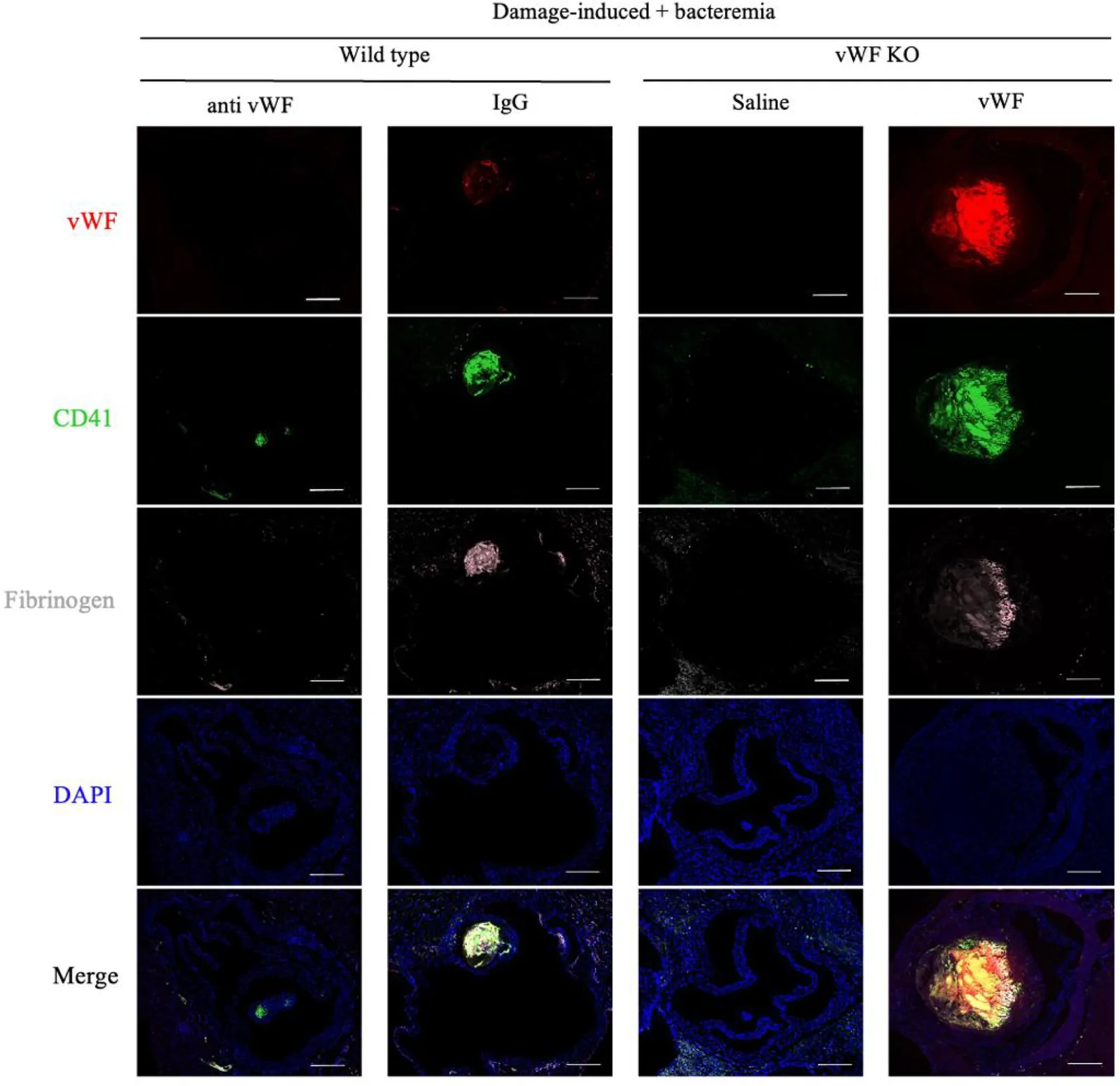
Immunofluorescence analysis of VWF manipulation effects on valve vegetation formation. Valve tissues were analyzed under conditions of VWF manipulation: Wild-type with anti-VWF treatment, Wild-type with control IgG, VWF KO with saline, and VWF KO with VWF supplementation. Tissues were stained for VWF (red), CD41 (green), fibrinogen (gray), and DAPI (blue). Overlay images are shown in the bottom row. VWF supplementation in VWF KO mice leads to substantial vegetation formation, characterized by co-localization of platelets, VWF, and fibrinogen. VWF blocking in wild-type mice prevents vegetation formation. Scale bars: 100 μm.

Immunofluorescence analysis of these manipulated conditions (Figure 11) provided further insights into the composition of the vegetations. VWF supplementation in VWF KO mice led to substantial vegetation formation, characterized by co-localization of platelets, VWF, and fibrinogen (Figure 11D). In contrast, VWF blocking in wild-type mice prevented the formation of organized vegetations, with only sparse platelet aggregates observed (Figure 11A).

### Inflammatory factor expression in wild-type and VWF knockout mice

**Figure 12:**
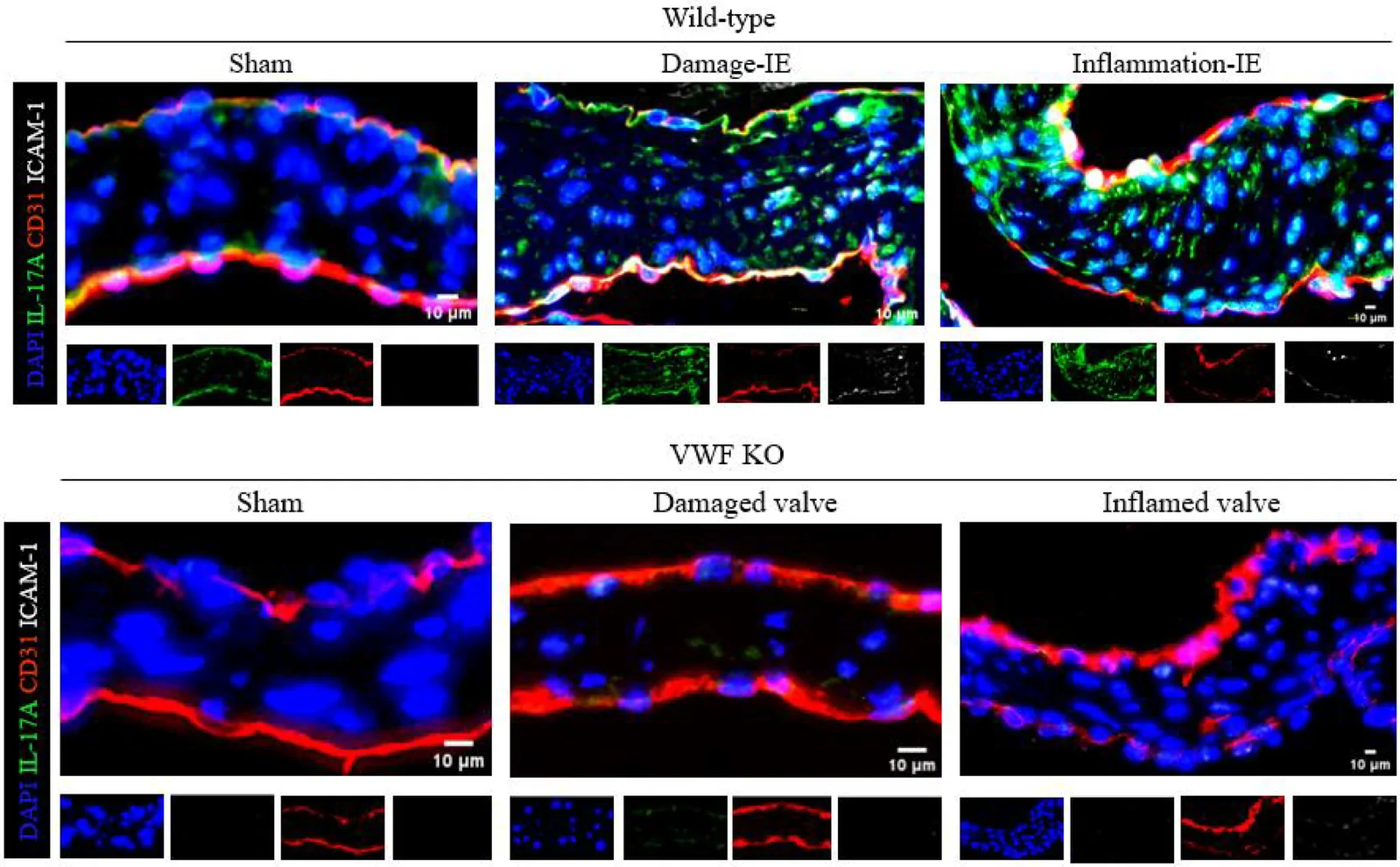
Inflammatory factor expression in wild-type and VWF knockout mice valve tissues under different endocarditis conditions. Valve tissues from wild-type and VWF knockout (KO) mice were analyzed under sham condition, damage-induced endocarditis (Damaged valve), and inflammation-induced endocarditis (Inflamed valve). Main images show overlay of DAPI (blue), IL-17A (green), CD31 (red), and ICAM-1 (white) staining. Individual channels are shown in smaller images below. VWF KO mice exhibit reduced expression of inflammatory factors in both endocarditis models compared to wild-type mice. Scale bars: 10 μm.

Analysis of inflammatory factor expression in valve tissues revealed significant differences between wild-type and VWF KO mice under different endocarditis conditions (Figure 12). Wild-type mice showed increased expression of IL-17A, CD31, and ICAM-1 in both damage-induced and inflammation-induced endocarditis models compared to sham conditions (Figure 12A–C).

In contrast, VWF KO mice exhibited reduced expression of these inflammatory factors in both endocarditis models (Figure 12D–F). This reduction was particularly pronounced in the damage-induced model, where VWF KO mice showed only modest increases in inflammatory marker expression compared to sham conditions.

### IL-17A/ICAM-1 acts downstream of VWF

*In vitro*, *S. mutans* infection significantly upregulated VWF, IL-17A, and ICAM-1 in HUVECs (all *P* < 0.05; Figure 13A–E). This induction was dose-dependently suppressed by anti-VWF neutralizing antibody: 10 μg/mL antibody reduced VWF by 72%, IL-17A by 68%, and ICAM-1 (mRNA/protein) by 57%/52% (all *P* < 0.05), with weaker inhibition at 5 μg/mL. Isotype control IgG had no effect (*P* > 0.05).

## Discussion

This comprehensive study provides novel insights into the distinct mechanisms of vegetation formation in damage-induced and inflammation-induced *Streptococcus mutans* endocarditis models, with a particular focus on the role of von Willebrand Factor (VWF). Our findings demonstrate that VWF plays a critical, yet differential role in the pathogenesis of endocarditis depending on the initiating mechanism.

### Distinct kinetics of vegetation formation

The histological and ultrastructural analyses revealed striking differences in the kinetics of vegetation formation between the damage-induced and inflammation-induced models. The damage-induced model exhibited rapid vegetation development, with significant thickening of valve leaflets observed as early as 24 h post-induction. This swift progression is likely due to the immediate exposure of subendothelial matrix proteins following mechanical injury, which provides an ideal substrate for platelet adhesion and bacterial colonization (8).

In contrast, the inflammation-induced model showed a more gradual progression of vegetation formation. This delayed response may be attributed to the time required for systemic inflammation to induce endothelial activation and dysfunction, eventually leading to exposure of adhesive proteins and subsequent vegetation formation (24). These findings underscore the importance of considering the initiating mechanism when studying endocarditis pathogenesis and evaluating potential therapeutic interventions.

### Temporal and spatial patterns of VWF involvement

Our analysis of plasma VWF:Ag levels and VWF mRNA expression in valve tissues revealed distinct temporal patterns between the two models. The rapid increase in plasma VWF:Ag levels observed in the damage-induced model suggests an immediate release of VWF from endothelial Weibel-Palade bodies in response to vascular injury (11). This acute release of VWF likely contributes to the rapid formation of platelet-rich vegetations observed in this model.

The delayed increase in VWF:Ag levels in the inflammation-induced model aligns with the more gradual progression of vegetation formation observed histologically. This pattern may reflect the time required for inflammatory mediators to stimulate *de novo* synthesis and release of VWF from endothelial cells (25). The significant upregulation of VWF mRNA expression in both models by day 5 suggests that sustained VWF production contributes to the progression and stabilization of vegetations over time.

Immunohistochemical and immunofluorescence analyses provided valuable insights into the spatial distribution of VWF within developing vegetations. The extensive co-localization of VWF with platelets and fibrinogen in both models, particularly in advanced stages, highlights the central role of VWF in vegetation architecture. These findings are consistent with previous studies demonstrating VWF's ability to form fiber-like structures that can trap platelets and promote thrombus growth (26).

### Critical role of VWF in vegetation formation

The experiments using VWF knockout mice provided compelling evidence for the essential role of VWF in vegetation formation, particularly in the damage-induced model. The striking reduction in vegetation size and complexity in VWF KO mice underscores VWF's importance in initiating and sustaining platelet adhesion and aggregation at sites of endothelial damage (27).

Interestingly, the inflammation-induced model in VWF KO mice still exhibited small vegetation formation, albeit significantly reduced compared to wild-type mice. This observation suggests that while VWF plays a dominant role in vegetation formation following direct endothelial damage, other adhesive proteins may partially compensate in its absence, particularly in the context of inflammation-induced endothelial activation (28).

The VWF manipulation experiments further solidified the causal relationship between VWF and vegetation formation. The ability of anti-VWF antibody treatment to inhibit vegetation development in wild-type mice suggests a potential therapeutic avenue for preventing or limiting endocarditis progression. Conversely, the restoration of vegetation formation in VWF KO mice following VWF supplementation confirms that VWF is not only necessary but sufficient to promote substantial vegetation growth in the presence of other required factors.

### VWF as a modulator of inflammatory responses

An intriguing finding of our study was the reduced expression of inflammatory markers (IL-17A, CD31, and ICAM-1) in VWF KO mice during endocarditis progression. This observation suggests that VWF may play a previously underappreciated role in modulating the inflammatory response during endocarditis.

Several mechanisms could explain this phenomenon. First, VWF has been shown to interact with leukocytes through P-selectin glycoprotein ligand-1 (PSGL-1), facilitating their recruitment to sites of inflammation (29). In the absence of VWF, this recruitment may be impaired, leading to a reduced inflammatory infiltrate. Second, VWF-platelet interactions have been implicated in the release of pro-inflammatory mediators from activated platelets (30). The reduction of these interactions in VWF KO mice may result in a dampened inflammatory response.

Additionally, the extensive fibrin and platelet-rich vegetations formed in the presence of VWF may serve as a nidus for ongoing inflammation, trapping bacteria and promoting sustained leukocyte activation (3). The reduced vegetation formation in VWF KO mice may thus indirectly lead to a less pronounced inflammatory response.

This study demonstrates that VWF mediates S.mutans-induced IL-17A secretion and ICAM-1 upregulation in endothelial cells, linking VWF to proinflammatory pathways in IE. The inhibition of IL-17A and ICAM-1 by anti-VWF antibody suggests that VWF may act upstream of these molecules, potentially via endothelial activation or leukocyte-endothelial crosstalk. The restoration of IL-17A and ICAM-1 expression by recombinant VWF confirms its specific role in this pathway.

IL-17A and ICAM-1 are critical for vegetation formation, as they promote leukocyte recruitment and bacterial sequestration in IE lesions. Our findings align with *in vivo* data showing that VWF deficiency reduces vegetation positivity, while VWF supplementation restores pathogenicity. Together, these results indicate that VWF contributes to IE pathogenesis partially through regulating IL-17A and ICAM-1.

By establishing S. mutans-specific IE models, we mechanistically linked VWF to vegetation formation via platelet adhesion and IL-17A/ICAM-1-driven inflammation. This extends beyond prior studies on S. aureus IE, where VWF primarily facilitates bacterial adhesion (22).

### Implications for endocarditis pathogenesis and therapy

Our findings hold important implications for elucidating the pathogenesis of infective endocarditis (IE) and developing novel therapeutic strategies. The distinct kinetics of VWF involvement in damage-induced vs. inflammation-induced IE indicate that the timing and nature of therapeutic interventions may need to be tailored to the underlying mechanism of endocardial injury. The ability of anti-VWF antibody treatment to suppress vegetation formation underscores the potential of VWF-targeted therapies in preventing or mitigating IE progression—an approach that could be particularly advantageous for high-risk patients undergoing procedures associated with endothelial damage.

Notably, species-specific differences between human and murine hearts cannot be overlooked (31). Morphologically, murine cardiac valves lack the characteristic three-layer architecture of human valves, with thinner leaflets that may alter VWF deposition and Streptococcus mutans adherence. Functionally, the higher murine heart rate (600–700 beats/min) elevates valvular blood flow velocity and shear stress, thereby modifying interactions between VWF multimers and valvular endothelial cells. At the molecular level, murine valvular endothelial cells selectively upregulate Icam2, whereas RNASE1 is a human-specific marker in this cell population—this molecular divergence may underlie differential valvular tissue responses to VWF-mediated inflammatory cues. Our murine model findings support the need for further investigation of VWF-targeted therapies in large animals（e.g., Porcine models）to bridge preclinical and clinical research.

The observed reduction in both vegetation formation and inflammatory marker expression in VWF KO mice suggests that VWF-targeted therapies may offer dual benefits by addressing both the thrombotic and inflammatory aspects of endocarditis. The distinct temporal patterns of plasma VWF:Ag levels observed in the two models suggest that VWF could serve as a biomarker for assessing the mechanism and stage of endocarditis progression, potentially guiding treatment decisions. Finally, the residual vegetation formation observed in VWF KO mice in the inflammation-induced model highlights the importance of considering potential compensatory mechanisms when developing targeted therapies.

While inhibiting VWF may suppress the development of infective endocarditis (IE) by reducing thrombus formation, it could also increase bleeding tendency, especially in patients with pre-existing coagulation disorders or those undergoing anticoagulant therapy. Anti-VWF antibody therapy (e.g., caplacizumab) seems to be with a higher incidence of bleeding-related adverse events compared to placebo (65% vs. 48%), raising concerns about bleeding risk in clinical application. However, the bleeding events are predominantly mild to moderate (mainly epistaxis and gingival bleeding), resolve spontaneously in most cases without additional intervention, and severe or serious bleeding is infrequent. Confirms the controllability of bleeding risks associated with anti-VWF antibody therapy (32).

### Limitations and future directions

While our study provides valuable insights into the role of VWF in endocarditis pathogenesis, several limitations should be acknowledged. Our experiments focused on *Streptococcus mutans*-induced endocarditis, and future studies should investigate whether the observed patterns hold true for other common endocarditis-causing pathogens. While mouse models offer many advantages for studying endocarditis, there are inherent differences in cardiac anatomy and hemodynamics compared to humans, and validation of key findings in larger animal models or human samples would strengthen the translational potential of this work. Although we examined multiple time points, more frequent sampling, particularly in the early stages of endocarditis development, could provide a more detailed understanding of the kinetics of VWF involvement. While our study demonstrates a clear role for VWF in vegetation formation and inflammation, further investigation into the molecular mechanisms underlying these effects is warranted. Lastly, while our VWF manipulation experiments suggest therapeutic potential, more extensive studies on the efficacy and safety of VWF-targeted interventions in preventing or treating endocarditis are needed. Future research directions stemming from this work could include investigating the interplay between VWF and other adhesive proteins in vegetation formation, exploring the molecular mechanisms by which VWF modulates the inflammatory response in endocarditis, developing and testing targeted anti-VWF therapies for endocarditis prevention or treatment, examining the potential of VWF as a biomarker for endocarditis progression and treatment response in clinical settings, and investigating the role of VWF polymorphisms or post-translational modifications in susceptibility to endocarditis or disease severity.

## Conclusion

In conclusion, our study demonstrates that von Willebrand Factor plays a critical, yet differential role in the pathogenesis of *Streptococcus mutans*-induced endocarditis depending on the initiating mechanism. The rapid involvement of VWF in vegetation formation following direct endothelial damage contrasts with its more gradual contribution in inflammation-induced endocarditis. VWF not only serves as a key structural component of vegetations but also modulates the inflammatory response during disease progression.

These findings enhance our understanding of the complex interplay between hemostatic and inflammatory processes in endocarditis pathogenesis and highlight VWF as a promising therapeutic target. Therapeutically, Targeting the VWF-IL-17A-ICAM-1 axis may represent a novel therapeutic strategy to inhibit vegetation formation in IE.

## Data Availability

The original contributions presented in the study are included in the article/Supplementary Material, further inquiries can be directed to the corresponding authors.
